# Spatial distribution and source apportionment of nitrogen in typical plain river networks and bacterial community response

**DOI:** 10.3389/fmicb.2025.1578657

**Published:** 2025-07-01

**Authors:** Aiju You, Qiaoxi Zheng, Pengcheng Yao

**Affiliations:** ^1^Zhejiang Key Laboratory of River-Lake Water Network Health Restoration, Hangzhou, Zhejiang, China; ^2^Zhejiang Institute of Hydraulics and Estuary (Zhejiang Institute of Marine Planning and Design), Hangzhou, Zhejiang, China; ^3^Department of Water Resources of Zhejiang Province, Hangzhou, Zhejiang, China

**Keywords:** source appointment, microbial diversity, nitrogen, stable isotope, plain river network

## Abstract

**Introduction:**

The Yubei plain river network (YPRN) is confined and has poor hydrodynamics, resulting in the accumulation of pollutants. Therefore, it is of great significance to explore the mechanisms by which different anthropogenic contamination sources-namely domestic, aquaculture, industrial, and agricultural-affect nitrogen content, as well as the composition of nitrifying, denitrifying, and other bacterial communities.

**Methods:**

This study determined δ^15^N and δ^18^O by bacterial denitrification, and quantitatively evaluate the contribution of pollution source through MixSIAR. And the changes of the bacterial community were analyzed through 16S rRNA gene sequencing.

**Results:**

The concentration of total nitrogen (TN) revealed a distinct spatial pattern, with the industrial area demonstrating the highest levels, followed closely by the aquaculture area and the domestic and agricultural areas. The stable isotope analysis delineated three dominant pollution source areas within the study region: i) an industrial pollution dominant area, accounting for 55% of the pollutant load; ii) a domestic pollution dominant area (39%); and iii) an aquaculture pollution dominant area (43%). The industry pollution samples demonstrated the highest TN concentrations and the lowest ${\text{NO}}_{3}^{-}$/TN ratio. Strong nitrification activity under high dissolved oxygen (DO) in the study area was investigated using stable isotope analysis. Proteobacteria, Bacteroidetes, and Desulfobacteria were the dominant bacterial phyla in the study area. Notably, *Malikia* species with nitrate-reducing capabilities were significantly more abundant in the industrially pollution area compared to the other pollution areas.

**Discussion:**

The diversity of nitrogen types characteristic of the domestic pollution area mediated bacterial selection pressures, favoring nitrogen cycling and amplifying functional gene abundance. This bacterial activity enhanced nitrogen cycle efficiency, ultimately reducing nitrogen concentrations. Bacterial analyses revealed marked divergence in both community composition and function across different pollution types. Particularly, ecological network analysis showed greater complexity and more network links in the aquaculture pollution area. Overall, the results revealed the impacts of different pollution sources on the ecological processes shaping river microbial communities and determined variations in bacterial diversity and nitrogen-cycling gene abundances.

## 1 Introduction

River ecosystems are crucial for maintaining ecological balance and biodiversity conservation (Shao et al., 2023). Nitrogen loading in riverine ecosystems has escalated, with total nitrogen (TN) and nitrate (${\text{NO}}_{3}^{-}$) constituting the predominant forms (Liang et al., 2023). The spatiotemporal distribution of nitrogen is affected by external pollution sources and various biological interactions (Kim et al., 2023). Unreasonable nitrogen input (from domestic, aquaculture, industrial, and agricultural wastewater) leads to the degradation of river water environments (Liu et al., 2018). Excess nitrogen in rivers drives eutrophication while compromising bacterial metabolic vitality (Cao et al., 2020). Nitrogen pollution discharge and species-species ecological relationships substantially reduce bacterial diversity in heavily polluted aquatic systems (Dai et al., 2023). Therefore, analyzing nitrogen pollution and bacterial distribution can effectively clarify the mechanisms by which pollution sources influence bacterial communities.

At present, pollution source apportionment methods predominantly include source-oriented approaches, which estimate pollution loads based on land use types and empirical data (Shih et al., 2016). However, traditional methods exhibit deficiencies in tracing hidden and similar pollution sources. Stable isotope signatures exhibit distinct source-dependent variations, enabling precise identification of nitrate pollution (Cao et al., 2023). ^15^N and ^18^O are considered key tools for tracking nitrogen sources and have been successfully applied in rivers, lakes, and groundwater (Romanelli et al., 2020; Bu et al., 2017; Hu et al., 2019). This methodology exploits pollution source-specific isotopic characteristics (Xue et al., 2009). Kohl et al. were the first to determine the contribution of fertilizer-derived ${\text{NO}}_{3}^{-}$ in the Sangamon River using ^15^N-${\text{NO}}_{3}^{-}$ (Kohl et al., 1971). Combined stable isotope analysis and the MixSIAR model revealed the distribution and sources of nitrate in Karst landscapes, with synthetic nitrogen (36.6%) and organic nitrogen (28.0%) identified as the predominant pollution sources (Jun et al., 2022). Commonly, TN in plain river networks is dominated by ${\text{NH}}_{4}^{+}$ and ${\text{NO}}_{3}^{-}$ (Xuan et al., 2020). The compositions of nitrogen-stable isotopes provide additional information for the identification of nitrogen sources (Zhang et al., 2022).

High-concentration emissions may impose a serious load on river ecosystems (Berger et al., 2022). Wastewater from different sources contains various components, and industrial (Khan et al., 2008), domestic, agricultural, and aquaculture wastewater (Conkle et al., 2008) are predominantly characterized by organic nitrogen, ${\text{NH}}_{4}^{+}$, TN, and nitrogen-containing toxic compounds, respectively. The physicochemical parameters of wastewater, such as pH, TN, and total phosphorus (TP), can affect the structure and composition of microbial communities (Liu et al., 2020a). N cycling, which depends on microbial activities, is modulated by specific functional groups (Zhang et al., 2023a). Pascual-Benito et al. revealed that excess nutrients may reduce aquatic species diversity (Pascual-Benito et al., 2020). However, an increase in species diversity may result from more niches created by pollutants (Wakelin et al., 2008). The concentration and composition of nitrogen affect the bacterial community and structure (You et al., 2023) and dominate microbial nitrogen cycling (Li et al., 2021) in the river. Proteobacteria can enhance denitrification capacity in enriched nitrogen environments (Wu et al., 2016). Chloroflexi demonstrate significant nitrite-oxidizing functionality under eutrophic conditions (Sorokin et al., 2012). Therefore, research on bacterial community changes contributes to a better understanding of the functions of special taxa in different environmental conditions.

This study is based on the hypothesis that bacterial community structure changes through environmental selection. The main pollution sources in the research area were analyzed using stable isotope tracing, and the research area was classified into industrial, domestic, and aquaculture pollution areas according to pollution source analysis. The effect of pollution sources on bacteria was evaluated by observing bacterial diversities under different pollution conditions (Burns et al., 2016). This study provides quantitative information on nitrogen pollution sources and ecological insights into bacterial responses to different pollution sources.

## 2 Materials and methods

### 2.1 Study area and sample collection

**Figure 1:**
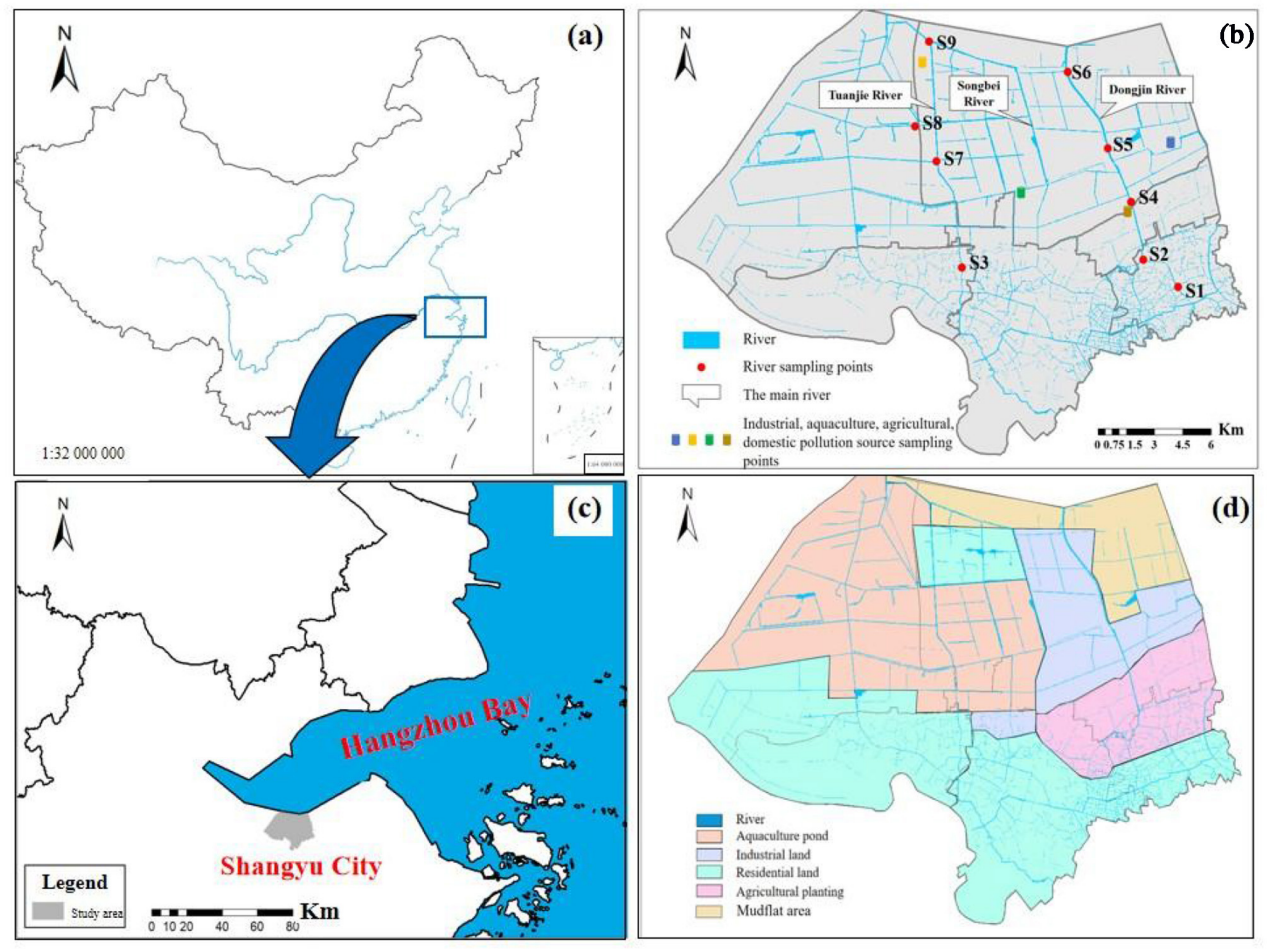
Sampling points and land-use types in the study area. **(a)** The location of study area. **(b)** The distribution of sampling points and river network in study area. **(c)** The distribution of study area. **(d)** Land-use types in the study area.

The Yubei plain river network (YPRN) in southern Hangzhou Bay was selected as the study area (Figures 1a, b). The research area covers 300 km^2^, with a total river network length of 200 km and a main river length of 25 km. In a comprehensive study of the Yubei Plain river network, nine spatially distributed sampling sites were selected from upstream to downstream, covering major land-use types such as aquaculture, industrial, residential, agricultural, and mudflat areas (Figures 1c, d). Detailed information on land-use types is provided in Supplementary Table S1. Land-use analysis delineated the research area into four functional units: industrial parks, agricultural planting areas, aquaculture areas, and residential gathering areas. The sampling sites were stratified by functional units: residential (S1 and S3), agricultural (S2), industrial (S4, S5, and S6), and aquaculture (S7, S8, and S9). These sampling sites typically represented the nitrogen pollution and bacterial community distribution in the study area. The nine samples, including surface sediments and their overlying water, were collected in May 2023. The sediments were collected using a grab sampler, and the water samples were collected using a water sampler. All collected samples were immediately transported to the laboratory at low temperatures.

To study the isotope characteristics of potential pollution sources such as industry, agriculture, aquaculture, and domestic areas, wastewater samples from various pollution sources were collected. Triplicate samples were collected at each pollution source, and all samples were analyzed for δ^15^N and δ^18^O. The physicochemical indicators of the sediment and water samples were tested three times repeatedly. All bacteria in the sediment samples were analyzed using 16S rRNA sequencing, and S2, S5, and S7 were selected for repeated sample testing. PCR and DNA extraction were carried out using ultrapure water as the blank sample. The test results showed that no DNA was detected in the blank sample.

### 2.2 Measurements of the environmental factors and stable isotopes

Dissolved oxygen (DO) and pH were measured using a multi-parameter water quality monitor (YSI6600 V2, USA) *in situ*. The water samples were filtered using 0.4 μm glass fiber membranes for detecting nitrate and stable isotopes, while the unfiltered water samples were used for detecting conventional water quality indicators such as total nitrogen (TN) and total phosphorus (TP). The detection of TP and TN was carried out using the alkaline potassium persulfate digestion-UV spectrophotometric method and the ammonium molybdate spectrophotometric method (UV-2450, Japan).

This study determined δ^15^N and δ^18^O via bacterial denitrification. In brief, N_2_O produced from ${\text{NO}}_{3}^{-}$-N and ${\text{NH}}_{4}^{+}$-N was quantified using an isotope ratio mass spectrometer (Delta V plus, Thermo Fisher Scientific, USA).

The stable isotope ratio was expressed using delta (δ) values as follows:


(1) $$\begin{array}{l} \delta_{\text{sample}}\left(\%\right)=\left(R_{sample}/R_{standard}-1\right)\times 1000 \end{array}$$

where R_sample_ and R_standard_ represent the ratios of ^15^N/^14^N and ^18^O/^16^O in the sample and standard, respectively. The reference standards for the ^15^N/^14^N and ^18^O/^16^O ratios are atmospheric air and Vienna Standard Mean Ocean Water, respectively.

### 2.3 MixSIAR receptor model

The MixSIAR model is a pollution source analysis tool based on the Markov chain Monte Carlo method and Bayesian framework. It combines the advantages of several models (IsoSource and SIAR) and has been successfully applied to quantify the contribution of pollution sources in rivers (He et al., 2022). The contribution proportion of potential pollution sources is based on the mass conservation of the tracer by the MixSIAR model. The equation is as follows:


(2) $$\begin{array}{l} X_{\text{ij}}=\sum_{k=1}^{ns}p_{ik}S_{kj}+\varepsilon_{ij} \end{array}$$

Where X_ij_ is the value of tracer j in mixture i; ns is the number of sources; P_ik_ indicates the contribution ratio of source k to mixture I; S_kj_ is the value of tracer j in pollution source k; and ε_*ij*_ is the error term for isotope j in mixture i (Stock et al., 2018).

MixSIAR was used to quantitatively evaluate the contribution of the pollution sources within the R 4.1.3 framework (Stock et al., 2018). In this study, δ^15^N and δ^18^O were used as tracers to assess the proportional contribution of each pollution source to nitrogen in river water.

### 2.4 DNA extraction and bacterial 16 S rRNA gene sequencing

In the study area, water flow within the YPRN is controlled by sluices and the water body is significantly disturbed by anthropogenic interference. Sediment stability can better characterize regional bacterial characteristics in the study area. Thus, bacteria in sediments were selected as the research focus of this study. DNA was extracted from 0.25 g of sediment using the PowerOil DNA Separation Kit (MoBio Laboratories, Carlsbad, CA, USA). The purity and concentration of the DNA were checked using a NanoDrop spectrophotometer (Nanodrop Technologies Inc., Wilmington, DE, USA). The V3–V4 region of the bacterial 16S rRNA gene was amplified using the primers 338 F (5′-ACTCCTACGGGAGGCAGCA-3′) and 806 R (5′-GGACTACHVGGGTWTCTAAT-3′) (Dennis et al., 2013). The adapters used for sequencing were as follows: 5′-AATGATACGGCGACCACCGAGATCTACACTCTTTCCCTACACGACGCTCTTCCGATCT-[insert]-AGATCGGAAGAGCACACGTCTGAACTCCAGTCAC[I7]ATCTCGTATGCCGTCTTCTGCTTG-3′ and 3′-TTACTATGCCGCTGGTGGCTCTAGATGTGAGAAAGGGATGTGCTGCGAGAAGGCTAGA-[insert]-TCTAGCCTTCTCGTGTGCAGACTTGAGGTCAGTG[I7]TAGAGCATACGGCAGAAGACGAAC-5′. PCR amplification was performed in a 20 μL reaction system containing a 5 × PCR buffer, 10 ng of DNA template, 0.25 mM of each dNTP, 0.2 μ*M* of each primer, and 1 U of FastPfu polymerase (TransGen, China; Shao et al., 2023). The PCR amplification process was as follows: initialization at 94°C for 3 min, 30 cycles of denaturation at 94°C for 30 s, annealing at 52°C for 30 s, extension at 72°C for 30 s, and final elongation at 72°C for 10 min (Chen et al., 2022). The purified PCR product was used to generate sequencing libraries using the NEBNext Ultra^TM^ DNA Library Prep Kit for Illumina (New England Biolabs, Ipswich, MA, USA). The species classification was based on the 16S rRNA database, and quality control of the raw data was completed using FastQC and KneadData. MEGAHIT and Kraken 2 were used to annotate microbial community sequences and species, respectively. Gene prediction and distribution analysis were performed using Prokka and Salmon (v0.13.1), respectively. The sequences have been uploaded to the NCBI Sequence Read Archive (SRA) database under accession number PRJNA1210818.

### 2.5 Statistical analysis of the bacterial community

The quality of the original sequence was evaluated using FastQC (v0.11.9), and quality control was performed using KneadData (https://github.com/biobakery/kneaddata). Clean reads were assembled using MEGAHIT (v1.2.9). Species annotation of the clean reads was performed using the Kraken 2 software, and gene pretesting of contigs was carried out using the Prokka software. The gene abundance table was generated using the Salmon (v0.13.1) software. FAPROTAX is a functional annotation database based on microbial classification used for the purpose of analyzing the functions of microbial communities. FAPROTAX assigns functions to species by comparing them with microbial functions previously reported in the literature. It has demonstrated relatively high accuracy in predicting microbial functions in marine and wetland environments. Therefore, FAPROTAX was selected to predict microbial functions in this study.

Statistical analyses were performed using R version 4.1.3. To visualize environmental gradients characterized by water physicochemical properties, principal component analysis (PCA) based on Euclidean distance was performed using the “prcomp” function from the “stats” package. Differences in bacterial community structures among areas with varying pollution levels were analyzed using non-parametric multivariate analysis (Adonis) and analysis of similarity based on functions in the “vegan” package.

Phylogenetic microbial ecological networks were constructed to infer potential interspecies relationships within the bacterial communities. Only OTUs detected in more than two-thirds of the samples were used for network construction for each of the three study areas to minimize the possibility of false positives. The correlation matrix was calculated using Spearman's rank correlation. Network modules were detected using fast greedy modularity optimization. Random networks corresponding to each empirical network were constructed by keeping the numbers of nodes and links constant and rewiring the nodes.

## 3 Results

### 3.1 Environmental factor concentration and spatial distribution

The TP concentration in the YPRN exhibited spatial variation, with average values of 0.150 mg/L, 0.554 mg/L, and 0.077 mg/L in the domestic and agricultural areas, industrial area, and aquaculture area, respectively (Supplementary Figure S1). According to the environmental quality standards for surface water (GB3838-2002, China), the TP concentrations in the domestic and agricultural areas, industrial area, and aquaculture area were classified as Class III, V, and II, respectively. The TP concentration in the industrial area exhibited significant elevation compared to other areas. DO and COD_Mn_ values in the YPRN ranged between 9 and 14 and 4 and 11.2 mg/L, respectively. A significant correlation was observed between COD_Mn_ and TP concentrations, indicating synchronous pollution patterns. The BOD_5_ value ranged from 3.4 to 5 mg/L, and the value of each area was similar.

**Figure 2:**
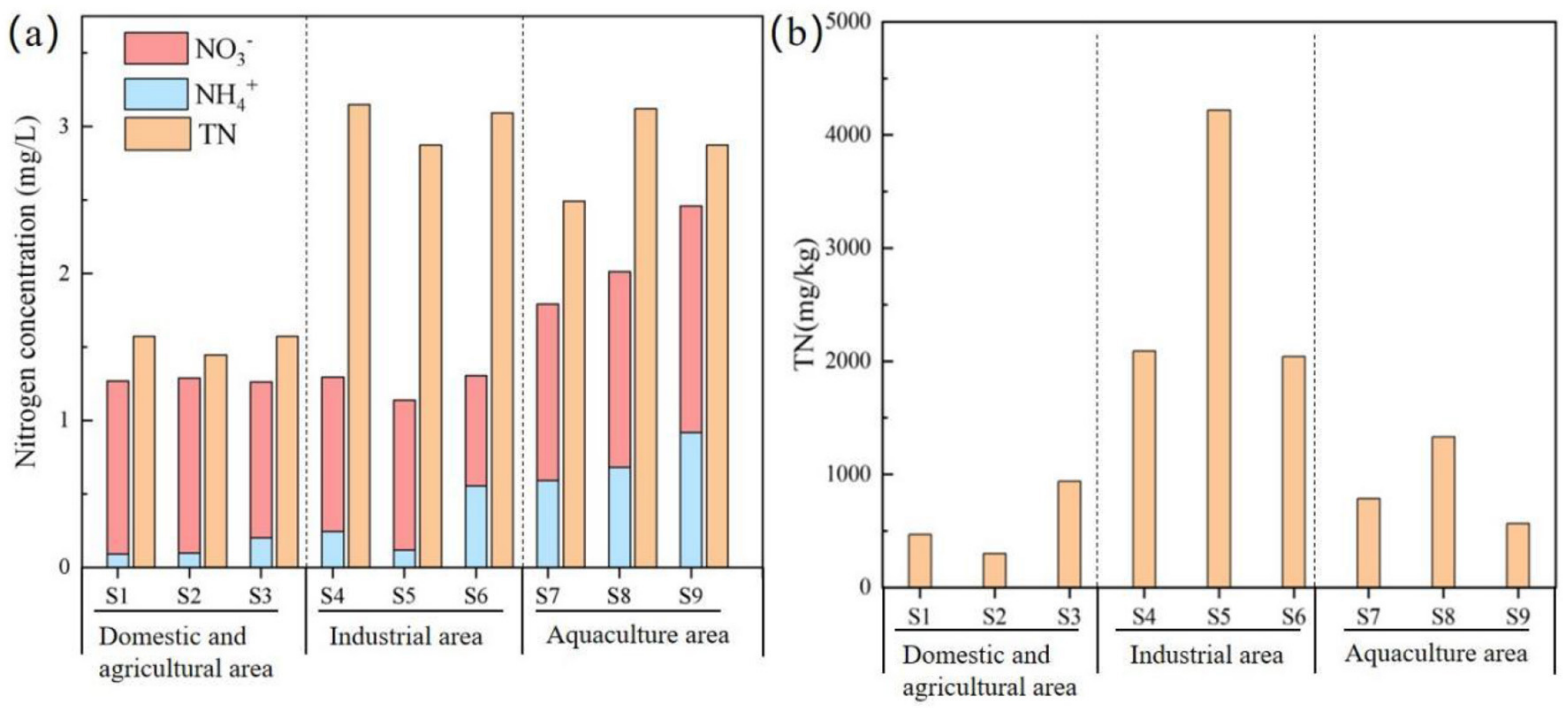
Distribution of nitrogen in the YPRN. **(a)** Overlying water; **(b)** Sediment.

The TN concentration in the YPRN ranged from 1.52 to 3.31 mg/L (Figure 2a), consistently exceeding Class V limits (GB3838-2002, China). The TN concentration showed significant heterogeneity, with the industrial area showing slightly higher concentrations than the aquaculture area and significantly higher levels observed in the domestic and agricultural areas. The ${\text{NO}}_{3}^{-}$ concentration ranged from 0.75 mg/L to 1.54 mg/L, which was significantly higher than the ${\text{NH}}_{4}^{+}$ concentration in the YPRN. Among the different areas, the ${\text{NO}}_{3}^{-}$/TN ratio was >64% in the domestic and agricultural areas, while it was ~30% in the industrial area. The TN concentration in the river sediment was between 299 and 4,220 mg/kg (Figure 2b). Sediment TN levels in the industrial area were significantly higher than those in the aquaculture, domestic, and agricultural areas.

### 3.2 Stable isotope distribution and nitrate source apportionment

δ^15^N and δ^18^O values in the YPRN ranged from 3.98‰ to 9.08‰ and from 3.29‰ to 6.93‰, respectively (Supplementary Figure S2). δ^15^N and δ^18^O values were highest in the domestic and agricultural areas and the aquaculture area, respectively, while both δ^15^N and δ^18^O values were lowest in the industrial area.

**Figure 3:**
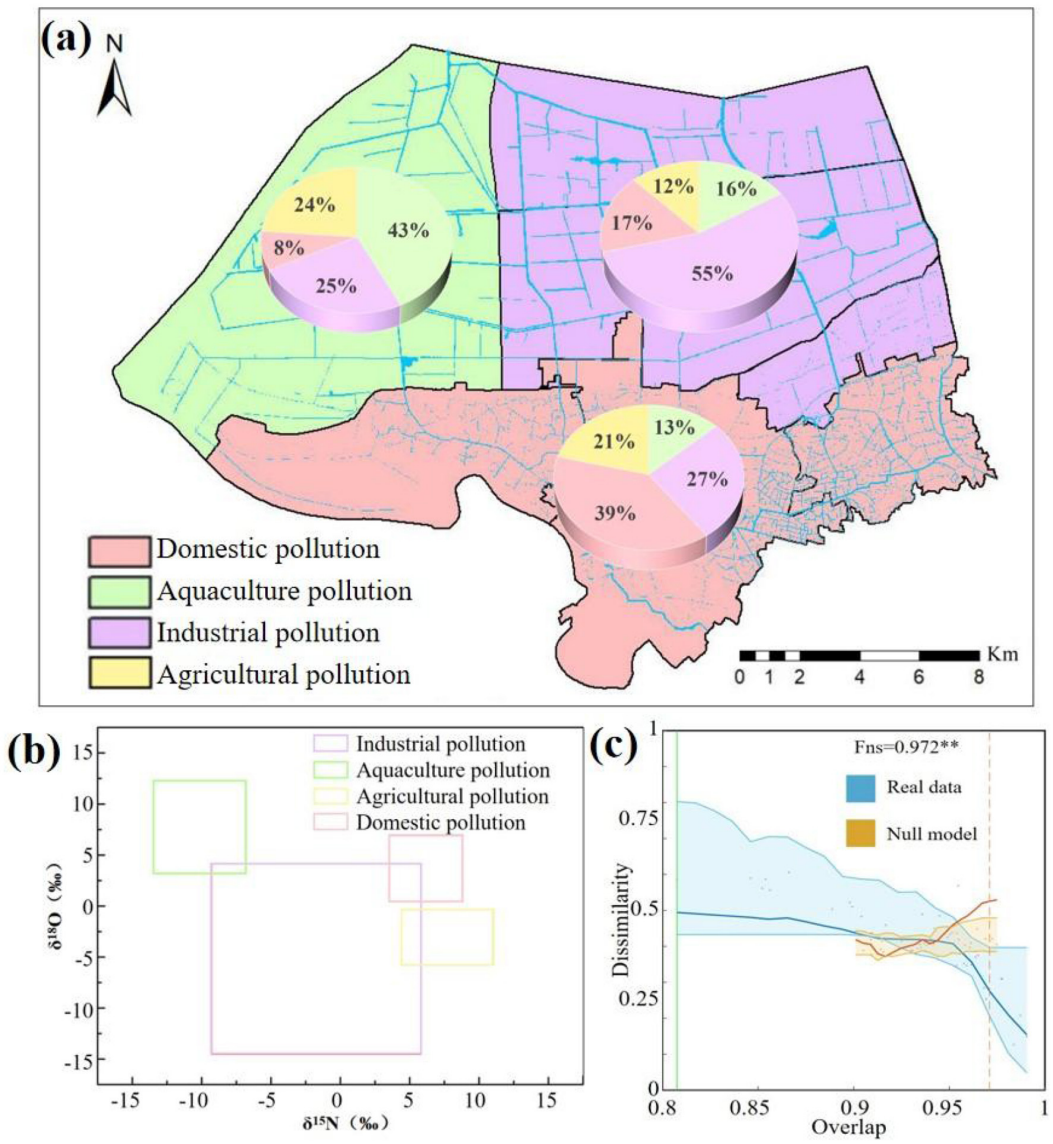
Contributions of the potential sources to nitrogen in the YPRN. **(a)** Result of the stable isotope analysis using MixSIAR; **(b)** Stable isotope values of the pollution sources; **(c)** DOC analysis of the sediment microbial communities. (In the DOC analysis, the horizontal axis and vertical axis represent the degree of competition among the bacteria and the dissimilarity between the microbial communities, respectively. Blue dots, yellow dots, and green lines represent the real values, the null model calculated values, and the change points, respectively).

δ^15^N and δ^18^O values were measured for each pollution source to improve the accuracy of source apportionment, and the results are shown in Figure 3b. The δ^15^N and δ^18^O values of the industrial pollution source ranged from −9.53‰ to 5.86‰ and −14.76‰ to 4.18‰, respectively. For the aquaculture pollution source, δ^15^N and δ^18^O values ranged between −13.97‰ and −6.34‰ and between 3.11‰ and 12.43‰, respectively. For the agricultural pollution source, δ^15^N values (3.35‰ −11.04‰) were significantly higher than the average values of the other pollution sources. The δ^18^O values of the agricultural pollution source were, on average, lower than those of the aquaculture and domestic pollution sources. For the domestic pollution source, δ^15^N and δ^18^O values ranged from 3.26‰ to 8.34‰ and from 0.53‰ to 6.07‰, respectively. The δ^15^N value was highest in the agricultural pollution source and lowest in the aquaculture pollution source, while the δ^18^O value was highest in the aquaculture pollution source and lowest in the industrial pollution source.

The MixSIAR framework incorporated four potential pollution sources (industry, agriculture, domestic, and aquaculture) to quantify nitrogen apportionment in the YPRN. Based on the land-use type analysis and source apportionment by MixSIAR, the study area was divided into three zones (Figure 3a): domestic pollution, industrial pollution, and aquaculture pollution. The MixSIAR results showed that the average nitrogen contribution in the YPRN upstream was 39% from domestic pollution, 27% from industrial pollution, 21% from agricultural pollution, and 13% from aquaculture pollution. According to the land-use type analysis, the YPRN downstream was mainly divided into industrial parks and aquaculture areas. Industrial pollution was the most dominant nitrogen source in the eastern downstream area of the YPRN, with a contribution of 55%, followed by domestic pollution (17%) and aquacultural pollution (16%). Nevertheless, aquacultural pollution became the largest nitrogen source in the western downstream area of the YPRN, followed by industrial pollution (25%) and agricultural pollution (24%).

Random ecological processes and environmental stress play pivotal roles in microbial community composition. The community dynamics of the YPRN were analyzed using the dissimilarity-overlap curve (DOC). The bacterial communities in the YPRN exhibited significant negative slopes in the DOC plot (Figure 3c). The extent of the negative slope in the DOC plot was quantified using the Fns value, and the Fns value showed a strong positive correlation with the degree of shared universal dynamics among the community. A null model was employed to simulate random ecological processes. There was a significant deviation between the observed data and the null model fit (Figure 3c), indicating the presence of non-random factors influencing community dynamics in the YPRN. The above results indicate that there are driving forces in the process of microbial community formation, especially environmental pressure.

**Figure 4:**
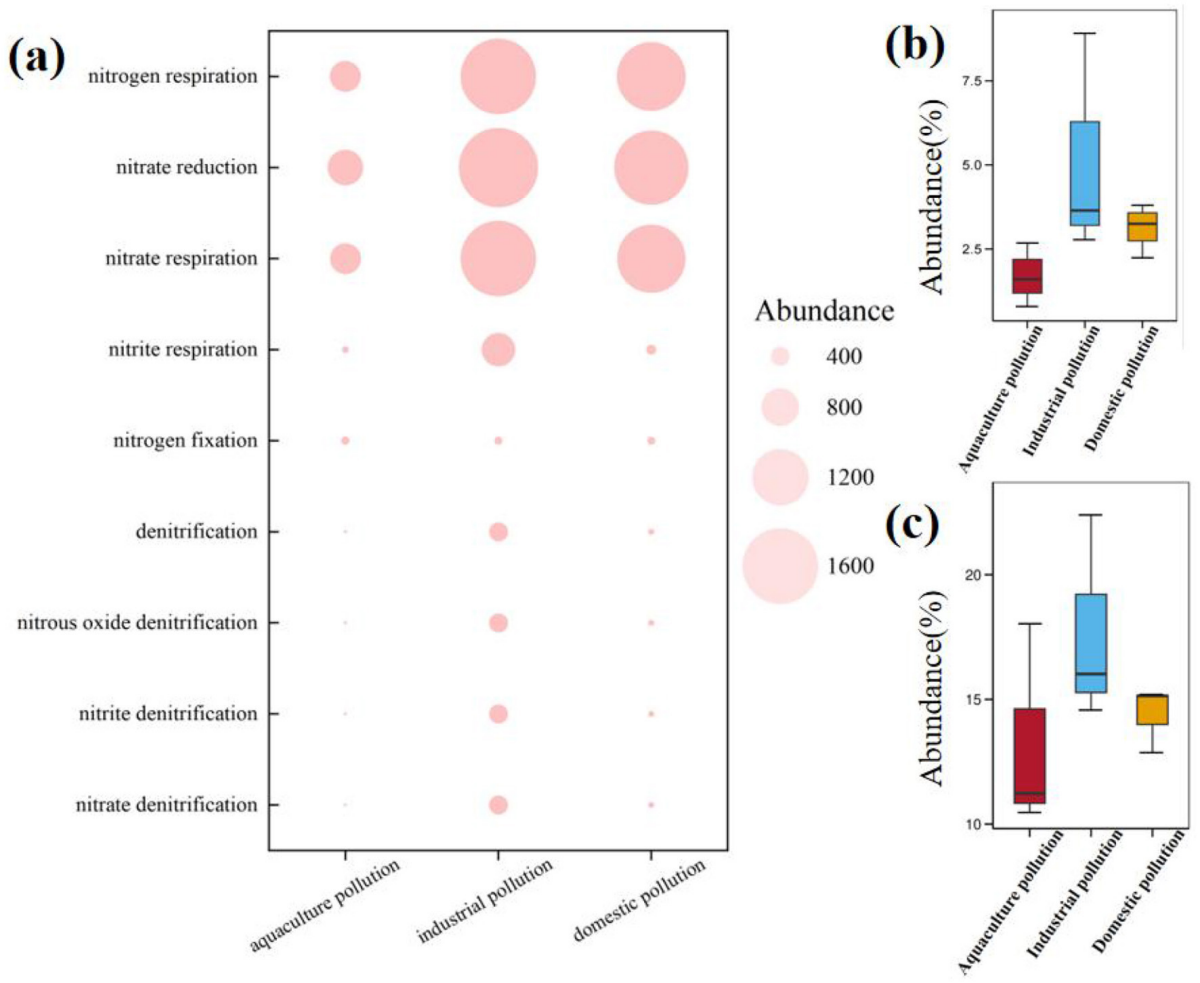
Abundance of the nitrogen cycle genes in samples from the industry pollution area, domestic pollution area, and aquaculture pollution area. **(a)** The abundance of nitrogen cycle genes in different pollution. **(b)** The abundance of nitrogen metabolism functions in different pollution. **(c)** The abundance of nitrate reduction in different pollution.

The high concentration of DO in the YPRN favored the occurrence of nitrification, and correlation analysis showed that DO was positively correlated with δ^15^N-${\text{NO}}_{3}^{-}$ (Supplementary Figure S4). A previous study reported that the δ^18^O-H_2_O value of the river ranged from −6.27‰ to −4.31‰ during nitrification, with theoretical values of δ^18^O-${\text{NO}}_{3}^{-}$ ranging from 3.65‰ to 4.96‰ (Torres-Martínez et al., 2020). These theoretical values were significantly lower than the δ^18^O-${\text{NO}}_{3}^{-}$ values observed in the YPRN (Supplementary Figure S3). Therefore, strong nitrification occurred in the river within the study area. As shown in Figure 2, the ratio of ${\text{NO}}_{3}^{-}$ to ${\text{NH}}_{4}^{+}$ in industrial and domestic pollution was significantly higher than in aquaculture pollution. The above results were closely related to DO concentrations (Supplementary Figure S4) and microbial functional genes (Figure 4a).

### 3.3 Bacterial community structure composition and diversity

**Figure 5:**
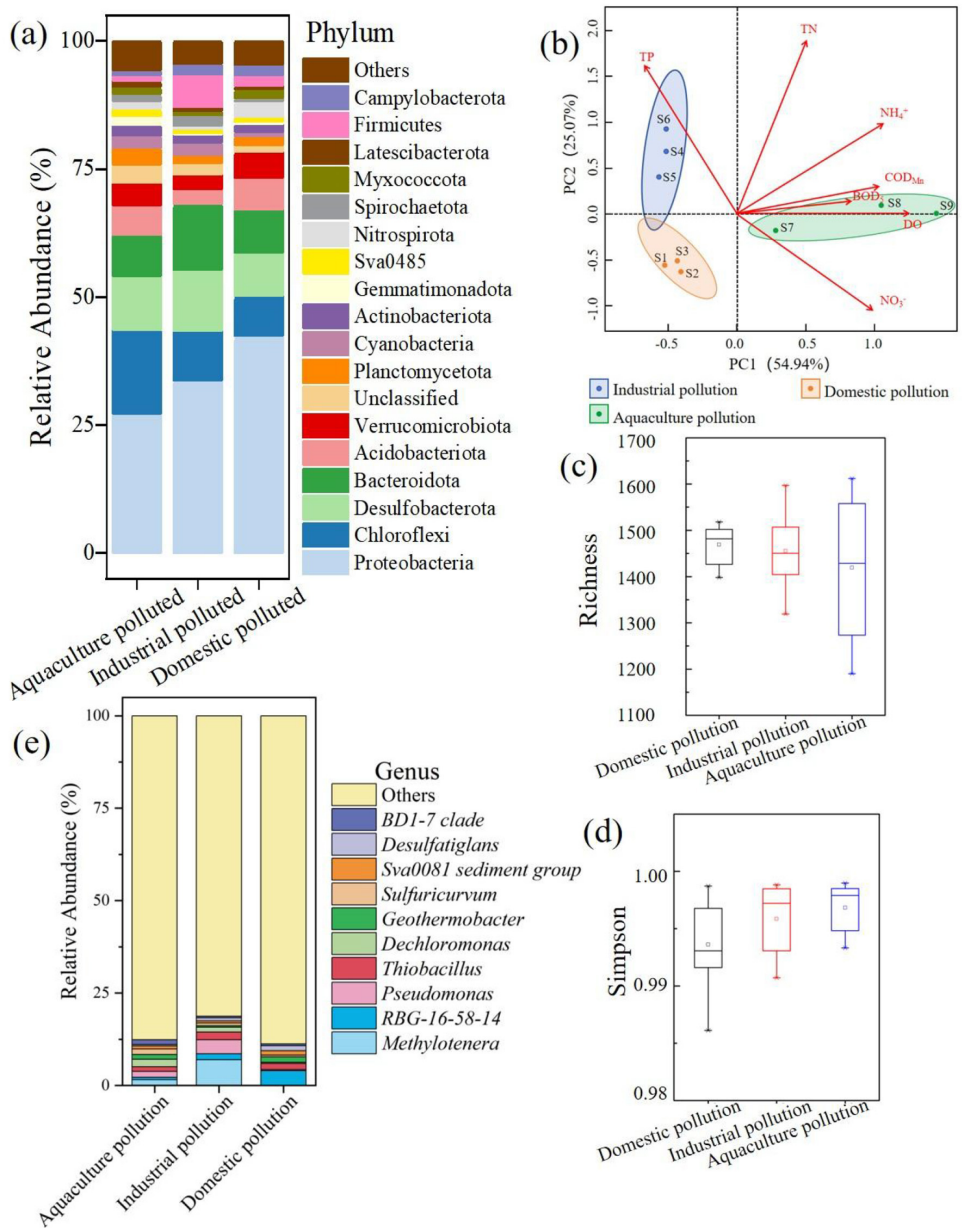
Bacterial community diversity and PCA biplot of the environmental factors. **(a)** The distribution of bacterial at the phylum. **(b)** The PCA analysis of bacterial. **(c)** The richness of bacterial in different pollution. **(d)** The simpson of bacterial in different pollution. **(e)** The distribution of bacterial at the genus.

The rarefaction curves indicated that the sequencing depth was sufficient to evaluate species diversity (Supplementary Figure S3). To identify differences in the bacterial communities within the study area, the classification and functional composition were grouped into industrial pollution, domestic pollution, and aquaculture pollution categories, as shown in Figure 3. Altogether, 75 microbial phyla were detected in the study area. Proteobacteria was the most abundant phylum across all groups at the phylum level (Figure 5a), followed by Bacteroidota, Desulfobacterota, and Chloroflexi. Among the dominant phyla, Proteobacteria showed significantly higher relative abundance in the domestic pollution group. Distinct pollution signatures emerged, with Chloroflexi predominating in the aquaculture pollution group and Bacteroidota characterizing the industrial pollution group. The alpha diversity indexes suggested low biodiversity and no significant differences in diversity among the groups in the study area (Supplementary Table S2). *Methylotenera* was the most abundant genus across all groups at the genus level (Figure 5e), followed by *RBG-16-58-14, Pseudomonas*, and *Thiobacillus*. Among these genera, *Methylotenera, Pseudomonas*, and *Thiobacillus* belong to the Proteobacteria phylum. The abundances of *Methylotenera* and *Pseudomonas* in the industrial pollution area were significantly higher compared to those in the other areas, and *RBG-16-58-14* was the dominant genus in the aquaculture pollution area.

Almost all factors related to nitrogen pollution (TN, ${\text{NH}}_{4}^{+}$, and ${\text{NO}}_{3}^{-}$) had high positive loadings on the first principal component (PC1; Figure 5b). The PC1 scores were highest for the samples from the aquaculture pollution area (Figure 5b). In contrast, TP displayed a negative loading on PC1 and a positive loading on PC2. The PC2 scores were highest for the samples from the industrial pollution area, suggesting that contaminant concentrations changed with the main pollutant contribution source. Simpson's diversity index reached its minimum in the domestic pollution area (Figure 5d), while richness was lowest in the aquaculture pollution area (Figure 5c). There were significant differences in the composition of the bacterial communities among the three areas.

### 3.4 Ecological networks

Based on the Spearman correlation analysis of the ecological networks, the two bacterial species were correlated across the sample sites (r_s_>0.95 or r_s_ < -0.95), and connection lines were drawn between the two nodes. In addition, ecological networks were constructed with OTU abundance correlation similarity thresholds of 0.85–0.93 (Supplementary Table S3), as determined automatically by RMT. The topological properties of the three empirical networks were different from those of the corresponding random networks (*p* < 0.05), suggesting that the empirical networks were statistically meaningful. The ecological networks showed a good fit (*R*^2^ = 0.75–0.96), indicating that they were scale-free and that the number of node links was moderate.

**Figure 6:**
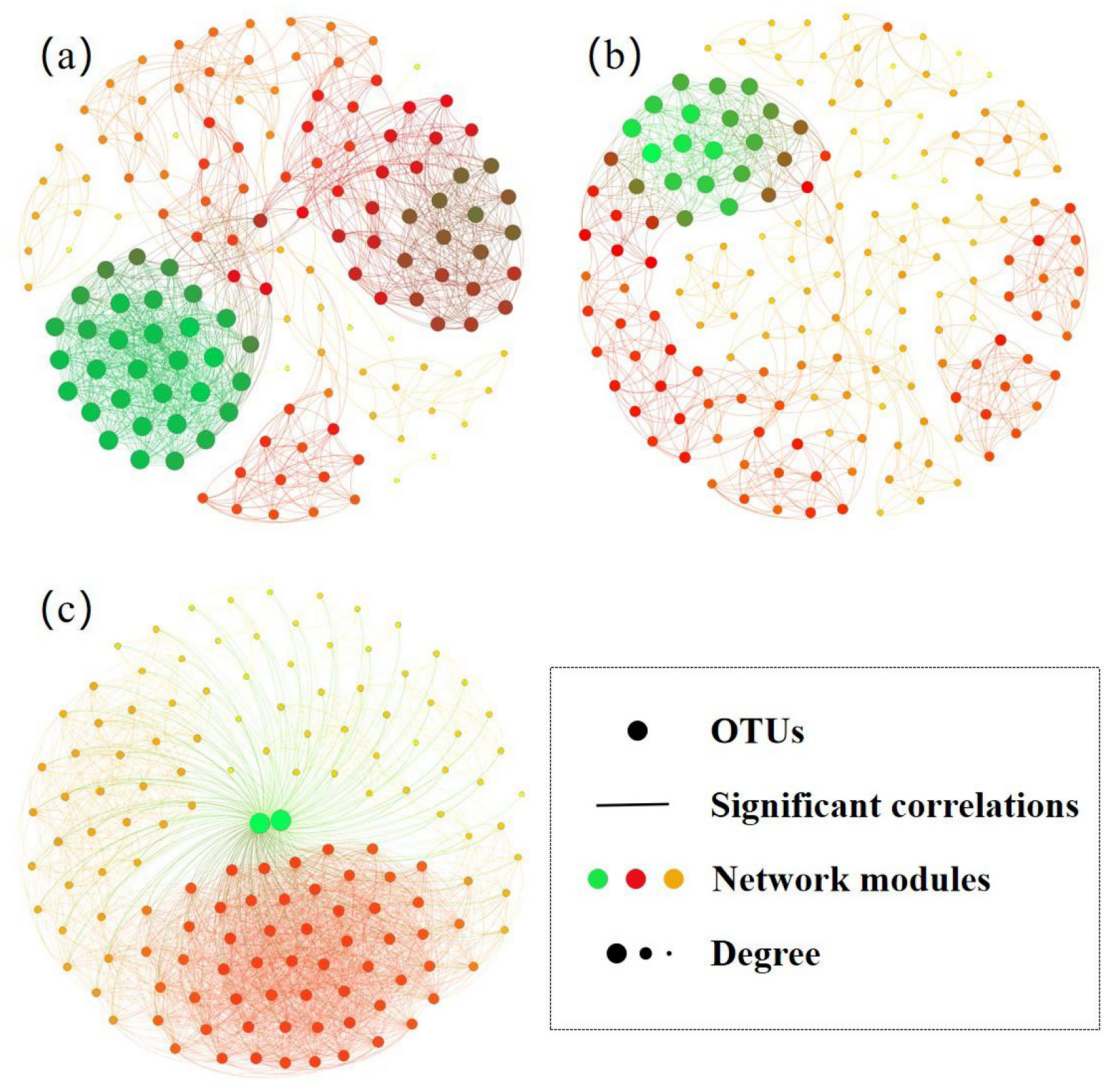
The ecological networks of the bacterial communities in the sediments. **(a)** industrial pollution; **(b)** domestic pollution; **(c)** aquaculture pollution.

Figure 6 shows that each node represents a microorganism, with red indicating a positive correlation and green indicating a negative correlation. The network size of domestic pollution (*n* = 171) was the largest compared to those of industrial pollution (*n* = 151) and aquaculture pollution (*n* = 138; Figure 6), which was consistent with the changes in OTU richness (Figure 5c). The node count, number of edges, average degree, connectivity, diameter, density, and modularity of the ecological network for the industrial pollution bacterial community were 151, 9,281, 122.93, 25, 2, 0.82, and 0.06, respectively. These parameters of the ecological network for the domestic and aquaculture pollution bacterial communities were as follows: 171, 8,857, 103.59, 75, 2, 0.61, and 0.20 and 138, 9,308, 134.90, 63, 2, 0.98, and 0.01, respectively. The ecological networks under aquaculture and domestic pollution consisted of several densely connected large modules and a few isolated small modules, whereas the network under industrial pollution exhibited higher modularity (Figure 6a). The ecological network analysis revealed that the ecological network in the industrial pollution area exhibited a significantly simpler topology, characterized by a higher proportion of negative correlations among bacteria. The ecological network in the aquaculture pollution area exhibited a more complex topology, characterized by fewer nodes but a greater number of links. In contrast, the ecological network in the industrial pollution area exhibited more nodes but fewer links, indicating a looser microbial structure. In addition, the ecological network in the industrial pollution area was disrupted under strong environmental pressure. In the industrial pollution area, competition among bacteria decreased, and bacteria formed more cooperative relationships to cope with highly polluted and toxic environments. The persistent influx of industrial pollutants imposed environmental pressure, fundamentally limiting the microbial community's capacity to develop complex and widely interconnected structures.

### 3.5 Microbial function prediction

Figure 4a illustrates distinct nitrogen metabolism functions across the pollution types, with industrial pollution and domestic pollution showing significantly higher activity than aquaculture pollution. Functional analysis showed that nitrogen respiration, nitrate reduction, and nitrate respiration genes were significantly higher than other functional genes. Quantitative functional profiling demonstrated an enrichment of nitrogen metabolism functions in industrial pollution, where nitrogen respiration (2.85%−8.14%; Figure 4b) and nitrate reduction (14.78%−23.11%; Figure 4c) levels significantly surpassed those in both aquaculture pollution and domestic pollution. The results confirmed that pollution sources can alter the bacterial potential for nitrogen metabolism. Industrial pollution stimulated nitrogen metabolism, whereas aquaculture pollution stressors suppressed these potential functions, reflecting different bacterial adaptation strategies to various pollution types.

## 4 Discussion

Strong anthropogenic stressors have significantly altered the land use situation within the study area in recent decades (Zhao et al., 2022). High population density, coupled with intensive agricultural, aquaculture, and industrial activities in the YPRN, has resulted in excessively high nitrogen loads in the region (Liu et al., 2018; Herbeck et al., 2021). High contribution of agricultural pollution to nitrogen levels is attributed to soil legacy nitrogen, which results from long-term excessive nitrogen application, with the average cropland nitrogen content reaching 1.45 g/kg (Zhou et al., 2020). The high concentration of nitrogen in aquaculture tailwater remains unaddressed (Zhou et al., 2022). Industrial operations tend to produce wastewater with high nitrogen concentrations, and the mismatch between low wastewater treatment standards and stringent water quality requirements has led to a significant contribution of industrial emissions to nitrogen pollution in the plain river network (Qin et al., 2022). Domestic sewage may still enter river networks through sewer infrastructure and runoff (Ji et al., 2022). Domestic, industrial, and aquaculture wastewater exhibit significant differences in composition, leading to distinct impacts on microbial community structures (Zhang et al., 2023b). Previous research has shown that Proteobacteria is the main phylum in rivers (Li et al., 2017) and that there is a significant correlation between Proteobacteria and nitrogen (Grube et al., 2012). This study revealed that bacteria diversity and taxa were higher in rivers impacted by domestic wastewater compared to those affected by other types of pollution. The increase in bacterial diversity was the cumulative effect of differences between domestic wastewater and river water bacterial communities (Wang et al., 2020), as well as the more homogeneous composition of domestic wastewater compared to other types of wastewater (Bian et al., 2024). Actinomycetes prefer to thrive in industrial wastewater containing polycyclic aromatic hydrocarbon compounds (Liu et al., 2007). Thus, Actinomycetes demonstrated enrichment in rivers impacted by industrial pollution, while their abundance was limited in rivers affected by other types of wastewater (Zhang et al., 2021).

Understanding the responses of bacterial structure and function to complex pollution sources is crucial. Romero et al. found that significant changes occurred in bacterial community structure when the influx concentration of ${\text{NO}}_{3}^{-}$ reached levels of 4.6–5.2 mg/L (Romero et al., 2019). In this study, more microbial functional genes related to nitrogen conversion were found in the industrial pollution area than in the domestic pollution area. The dominant bacterial species (such as Proteobacteria, Bacteroidota, and Chloroflexi) in industrial wastewater (Jiang et al., 2023) activate most steps of the nitrogen cycle, and high nitrogen concentrations can easily promote the transformation of bacterial community structure and function (Ruprecht et al., 2021). *Malikia* was matched with nitrogen cycling genes, including those involved in denitrification and nitrate reduction, which linked core individual taxa with putative functions (Banerjee et al., 2018). *Malikia* was significantly correlated with nitrate reduction (Zhang et al., 2020; Liu et al., 2020b) and preferred to thrive in nitrogen-enriched environment (Stokholm-Bjerregaard et al., 2017). In this study, the abundance of *Malikia* was highest in the industrial pollution area, and the abundance of nitrate reduction and nitrate respiration genes was significantly higher in the industrial pollution area compared to the other pollution areas.

Network modules have been interpreted as function units (Zhou et al., 2011), and lower modularity may reflect a dominant functional composition under strong selective pressure. The network size and modularity related to network complexity were significantly lower in the industrial pollution area compared to the other pollution areas. More isolated and smaller network modules reflect disrupted exchanges of material, energy, and information. According to the stress gradient hypothesis, negative interactions occur more frequently in eutrophic environments. Higher proportions of negative network links in the industrial pollution area reflect lower phylogenetic diversity. As negative network correlations can help balance bacterial communities (Yuan et al., 2021), the bacterial communities in the industry pollution area may be less stable to environmental perturbations than those in other pollution areas.

## 5 Conclusion

The contribution of different pollution sources to nitrogen in the YPRN was quantified using stable isotope analysis. We also analyzed the bacterial communities in the YPRN under different pollution conditions. The study found significant differences in the composition and function of bacterial communities under different pollution conditions. The differences in bacterial community diversity were not significant under different pollution types, and bacterial diversity affected by domestic pollution was slightly higher than that under other pollution types. The greater negative effect of aquaculture and industrial pollution highlights the importance of efficient wastewater treatment systems. Bacteroidota and Firmicutes showed the highest abundance in the industrial pollution area, and the abundance of functional genes related to nitrogen transformation was significantly higher in the industrial pollution area compared to the other areas. With continuous exposure to various pollution sources, increasing survey data on microbial diversity and function is an important step toward better understanding and predicting human-induced ecosystem changes.

## Data Availability

The original contributions presented in the study are publicly available. This data can be found at: https://www.ncbi.nlm.nih.gov/sra, accession number: PRJNA1210818.
