# Supplementary material for: Spatial distribution and source apportionment of nitrogen in typical plain river networks and bacterial community response
Source: Front Microbiol. 2025 Jul 1;16:1578657. doi: 10.3389/fmicb.2025.1578657 (PMC12259669; doi:10.3389/fmicb.2025.1578657)
Supplement: Supplementary file 1 [file Table_1.DOCX]

**Supporting Information**

***for***

**Spatial distribution and source apportionment of** **nitrogen in typical** **plain river networks and** **bacterial community response**

a Zhejiang Key Laboratory of Rivers and Lakes Water Grid Ecological Restoration, Hangzhou Zhejiang, 310000, China

b Zhejiang Institute of Hydraulics and Estuary (Zhejiang Institute of Marine Planning and Design), Hangzhou Zhejiang, 310000, China

c Department of Water Resources of Zhejiang Province, Hangzhou Zhejiang, 310000, China

# Table S1 Land-use classification of Yubei plain river network

| **Number** | **Region** | **Land-use classification** | **Area**  **(km^2^)** | **Description** |
| --- | --- | --- | --- | --- |
| 1 | Hangzhou  Bay industrial park | Industry, Domestic | 37.45 | Industrial production and supporting living areas |
| 2 | Lihai industrial park | Industry | 175 | Industrial production |
| 3 | Aquaculture area | Aquaculture | 103.93 | Aquaculture |
| 4 | Farmland village area | Agriculture, domestic | 58.06 | Village、farmland |
| 5 | Mixed agricultural area | Agriculture, domestic | 66.78 | Farmland, aquaculture, livestock, village |
| 6 | Greenhouse growing area | Agriculture, domestic | 23.00 | Viticulture |
| 7 | Coastal town | Domestic | 12.62 | Schools, domestic, businesses |
| 8 | Shoal area | Shoal | 37.04 | In idle |

# Table S2 The α-diversity of microbial communities in YPRN using ANOVA.

| **Group** | **Site** | **Simpson** | **Shannon** | **Pielou** | **Chao-1** |
| --- | --- | --- | --- | --- | --- |
| Domestic pollution | S1 | 0.996 | 7.055 | 0.833 | 5714.34 |
|  | S2 | 0.997 | 7.078 | 0.838 | 5578.05 |
|  | S3 | 0.998 | 7.354 | 0.858 | 6173.13 |
| Industrial pollution | S4 | 0.998 | 7.168 | 0.840 | 6201.14 |
|  | S5 | 0.998 | 7.125 | 0.837 | 6170.84 |
|  | S6 | 0.987 | 6.081 | 0.742 | 4600.38 |
| Aquaculture pollution | S7 | 0.999 | 7.401 | 0.876 | 5115.86 |
|  | S8 | 0.997 | 6.982 | 0.833 | 5073.48 |
|  | S9 | 0.997 | 7.192 | 0.838 | 6323.06 |

# Table S3 Topological properties of the empirical network and random networks.

| Network topological properties | Domestic pollution | |  | Industrial pollution | |  | Aquaculture pollution | |
| --- | --- | --- | --- | --- | --- | --- | --- | --- |
|  | Empirical network | Random networks (100 times) |  | Empirical network | Random networks (100 times) |  | Empirical network | Random networks (100 times) |
| Similarity threshold | 0.90 |  |  | 0.93 |  |  | 0.85 |  |
| R^2^ of power law | 0.75 |  |  | 0.96 |  |  | 0.86 |  |
| Average clustering coefficient (avgCC) | 0.77 | 0.46 |  | 0.91 | 0.57 |  | 0.99 | 0.62 |
| Centralization of betweenness (CB) | 0.0015 |  |  | 0.00095 |  |  | 0.000079 |  |
| Density (D) | 0.61 |  |  | 0.82 |  |  | 0.98 |  |
| Modularity(fast_greedy) | 0.20 | 0.10 |  | 0.062 | 0.034 |  | 0.0041 | 0.0051 |


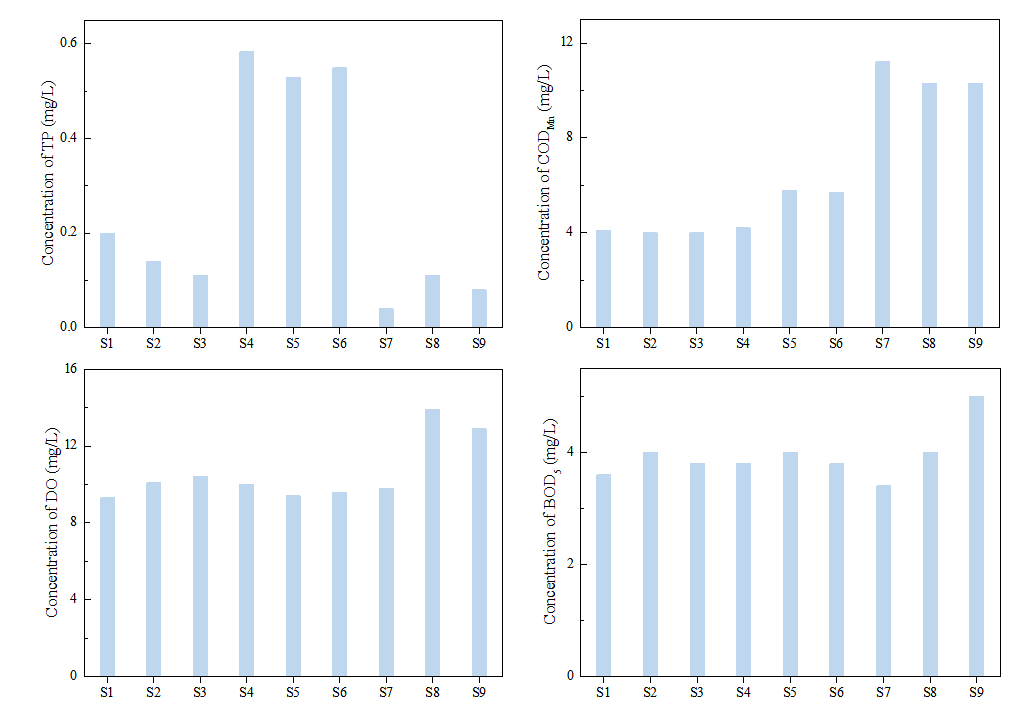


# Figure S1 Distribution of physicochemical indexes of YPRN.


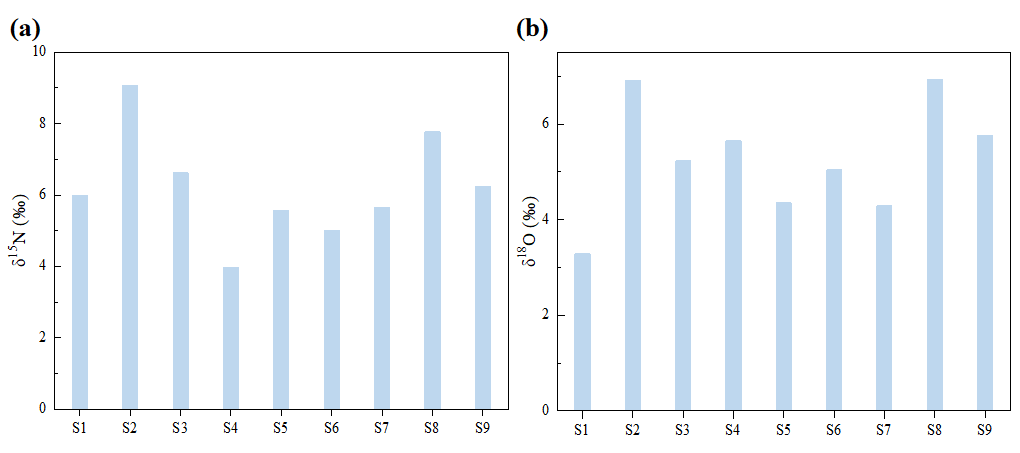


# Figure S2 Distribution of stable isotope (δ15N and δ18O) of YPRN.


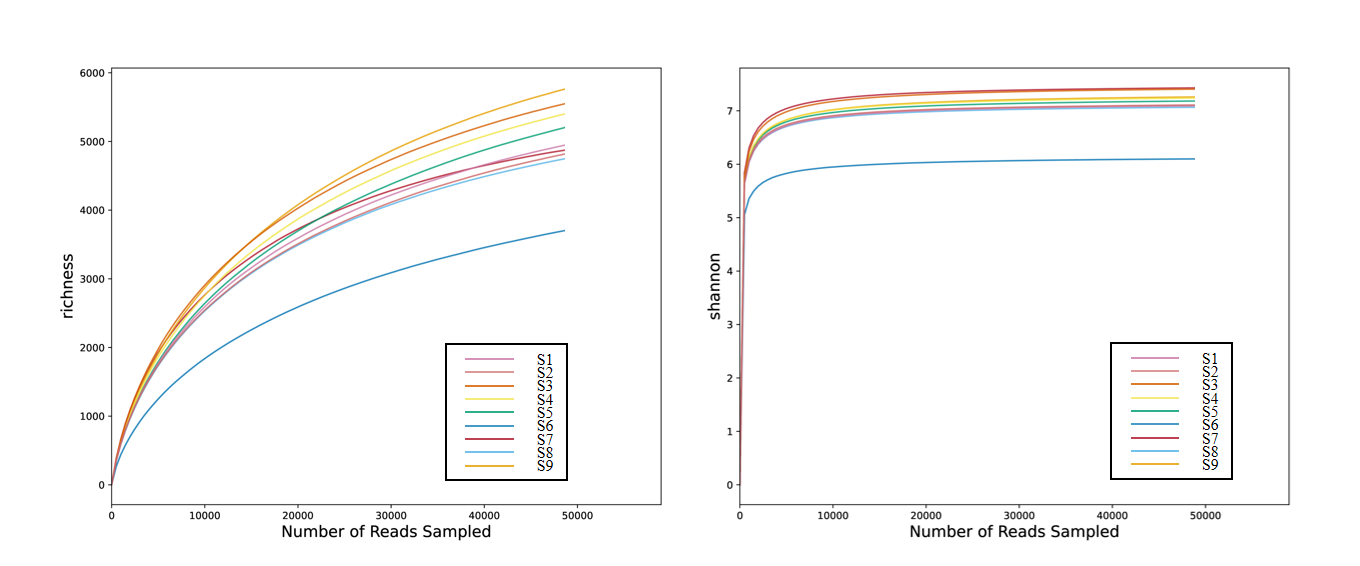


# Figure S3 Rarefaction curves showing the sequence efforts.


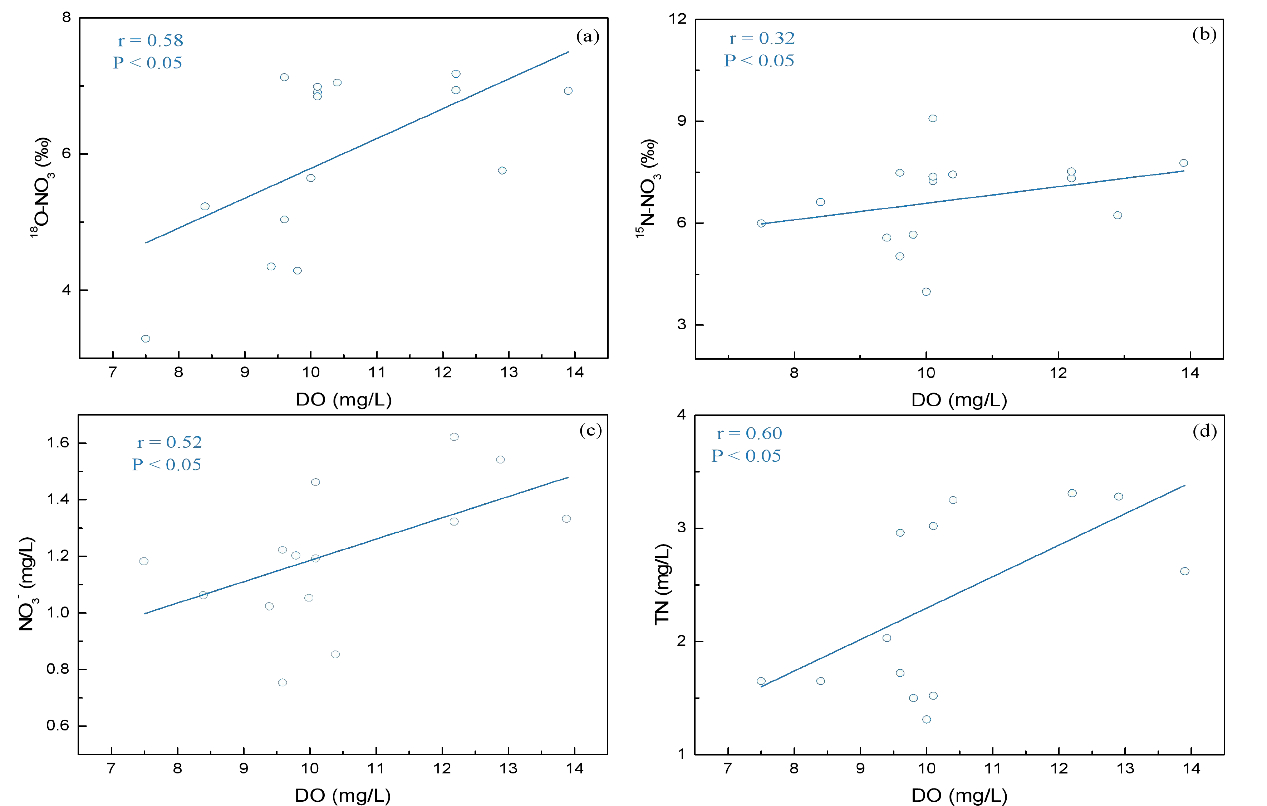
Figure S4 The relationship between DO and isotopic variables in the Yubei plain river network.


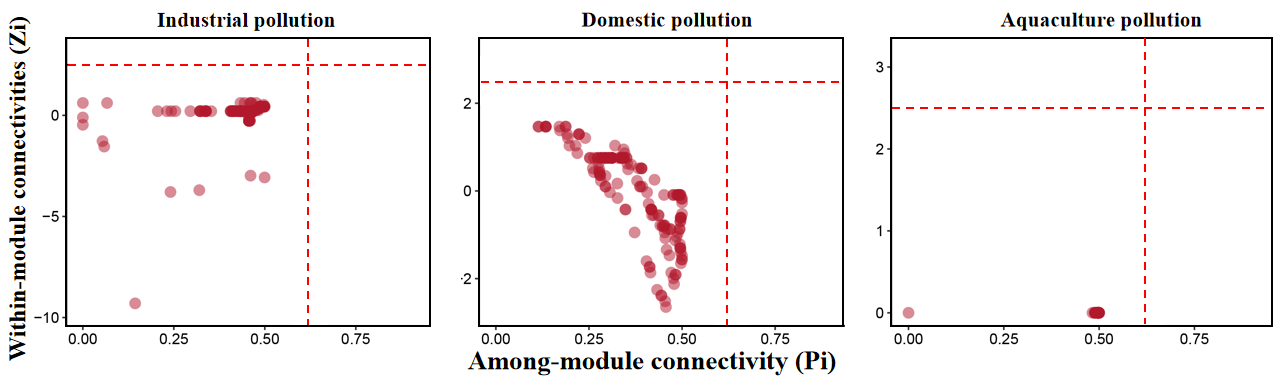


# Figure S5 The distribution of OTUs based on their within-module connectivity (Zi) and among module connectivity (Pi) in the networks.


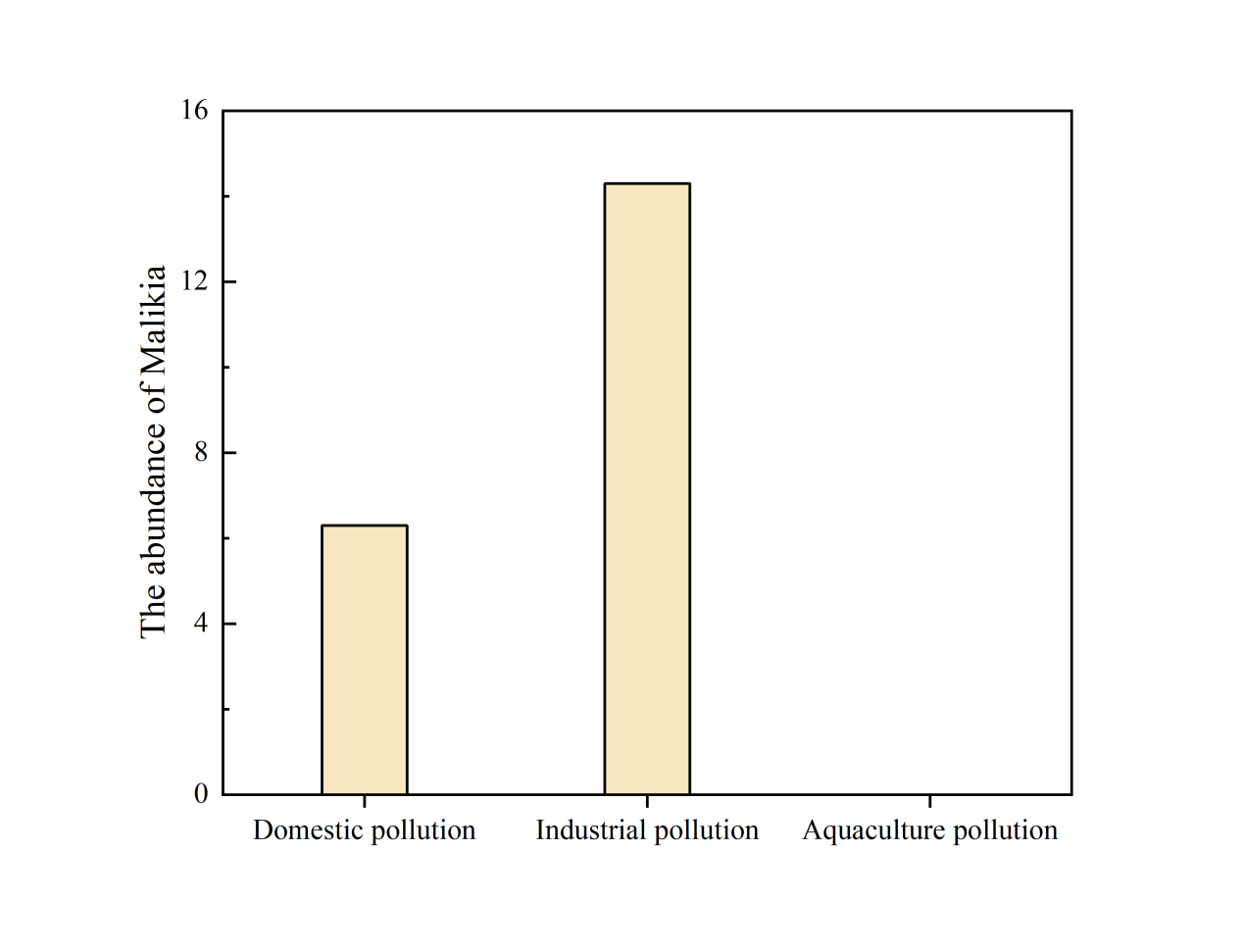


# Figure S6 The abundance of Malikia in YPRN.
